# HOG Signaling: A Multifunctional Regulator of Stress Homeostasis in *Saccharomyces cerevisiae*

**DOI:** 10.3390/jof12090668

**Published:** 2026-09-04

**Authors:** Mengmeng Ren, Xutong Xu, Ziying Wang, Hui Ma, Jinhai Wu, Jianping Guo

**Affiliations:** College of Food Science, Shanxi Normal University, Taiyuan 030031, China; xuxutong1021@163.com (X.X.); wzy20032023@163.com (Z.W.); huima@sxnu.edu.cn (H.M.); wujh@sxnu.edu.cn (J.W.)

**Keywords:** HOG pathway, stress adaptation, *Saccharomyces cerevisiae*

## Abstract

During growth and metabolism, the budding yeast *Saccharomyces cerevisiae* is continuously exposed to diverse environmental stresses, and conserved mitogen-activated protein kinase (MAPK) cascades execute core functions in stress sensing and adaptive regulation. The high-osmolarity glycerol (HOG) pathway, a classical MAPK cascade in *S. cerevisiae*, was initially identified as the core regulator of hyperosmotic stress responses. Over recent decades, accumulating evidence has revealed that the HOG pathway is not merely an osmoregulatory module but a versatile signaling hub that integrates multiple stress inputs and orchestrates a broad spectrum of adaptive responses. This review systematically summarizes the core architecture of the HOG pathway, including its upstream sensing branches (Sln1 and Sho1) and the conserved three-tiered MAPK cascade, with an emphasis on how different stressors engage distinct branches and lead to differential Hog1 phosphorylation kinetics. This review further discusses the multifaceted roles of the HOG pathway in stress adaptation, covering transcriptional reprogramming, cell cycle arrest, metabolic reprogramming centered on glycerol synthesis, and emerging functions such as cell wall remodeling, flocculation, mitophagy, and cross-talk with other MAPK pathways. By integrating classical and contemporary findings, this review presents a comprehensive view of the HOG pathway in *S. cerevisiae* and provides a reference for future research on stress signaling and engineering of this model organism.

## 1. Introduction

During growth, cells are frequently subjected to various unpredictable physical or chemical stresses, such as high temperature, oxidative stress, hyperosmotic stress, and toxic chemicals. To rapidly sense stress signals, activate damage repair mechanisms, and adapt to environmental changes, cells have evolved complex signal transduction networks that regulate related gene expression and metabolic reprogramming. A key example is the mitogen-activated protein kinase (MAPK) pathway, a highly conserved signaling cascade in eukaryotes. It transmits external stimuli through sequential phosphorylation of a three-tiered kinase cascade, ultimately regulating physiological processes such as transcription factor activation, cell cycle progression, and metabolic homeostasis, thereby mediating adaptive responses to external environments [1].

*S. cerevisiae* possesses multiple MAPK pathways that regulate distinct biological processes, including mating, cell wall integrity, and sporulation. Among these, the high-osmolarity glycerol (HOG) pathway, mediated by the Hog1 kinase, was initially identified for its critical role in osmotic homeostasis. Accumulating recent evidence has progressively revealed that this pathway also exerts key regulatory functions in diverse stress responses, including oxidative stress, acetic acid stress, heat shock, endoplasmic reticulum (ER) stress, and tolerance to toxic agents [2,3]. Several excellent reviews have previously covered the HOG pathway from osmotic and signaling perspectives, while Blomberg specifically examined glycerol metabolism in yeast osmoregulation [4,5,6,7]. Nevertheless, these works do not fully integrate the most recent mechanistic advances and do not systematically cover the expanding functional scope of Hog1 beyond osmoregulation. For example, the non-canonical activation of Hog1 via stress granules, the Hog1-dependent positive feedback loop on Pbs2, and the differential dephosphorylation of Pbs2 by PP2C phosphatases have only been elucidated recently and are therefore not covered in earlier reviews. Moreover, emerging roles in cell wall remodeling, flocculation, and mitophagy have largely been treated as separate topics rather than integrated into a unified framework.

This review provides a comprehensive synthesis of recent advances in HOG signaling in *S. cerevisiae*, covering both the molecular basis of stress-specific activation and the diverse physiological functions of Hog1. We first summarize the core architecture of the HOG pathway, including its upstream sensing branches and the conserved MAPK cascade, and then discuss its multifaceted roles in transcriptional reprogramming, cell cycle control, glycerol metabolism, and emerging functions such as cell wall remodeling, flocculation, mitophagy, and cross-talk with other MAPK pathways. This review is intended to serve as a useful reference for future studies on stress signaling and the engineering of robust yeast strains.

## 2. Core Structure and Regulatory Framework of the HOG Pathway in *Saccharomyces cerevisiae*

### 2.1. The Core MAPK Module: A Conserved Three-Tiered Kinase Cascade

The core MAPK module of the HOG pathway is an evolutionarily conserved three-kinase cascade, organized in the order of MAPKKK→MAPKK→MAPK [6]. Upon sensing extracellular or intracellular signals including hyperosmotic stress, the upstream MAPKKK undergoes phosphorylation and subsequently activates its corresponding MAPKK. The activated MAPKK then phosphorylates conserved residues in the activation loop of the downstream MAPK, thereby triggering its functional activation. In the classical HOG pathway, Hog1 functions as the core MAPK governing osmotic adaptation, while Pbs2 acts as the MAPKK specifically responsible for activating Hog1, functioning as a pivotal hub in signal transduction between upstream and downstream components [8].

### 2.2. Upstream Sensing Branches: Sln1 and Sho1

**Figure 1:**
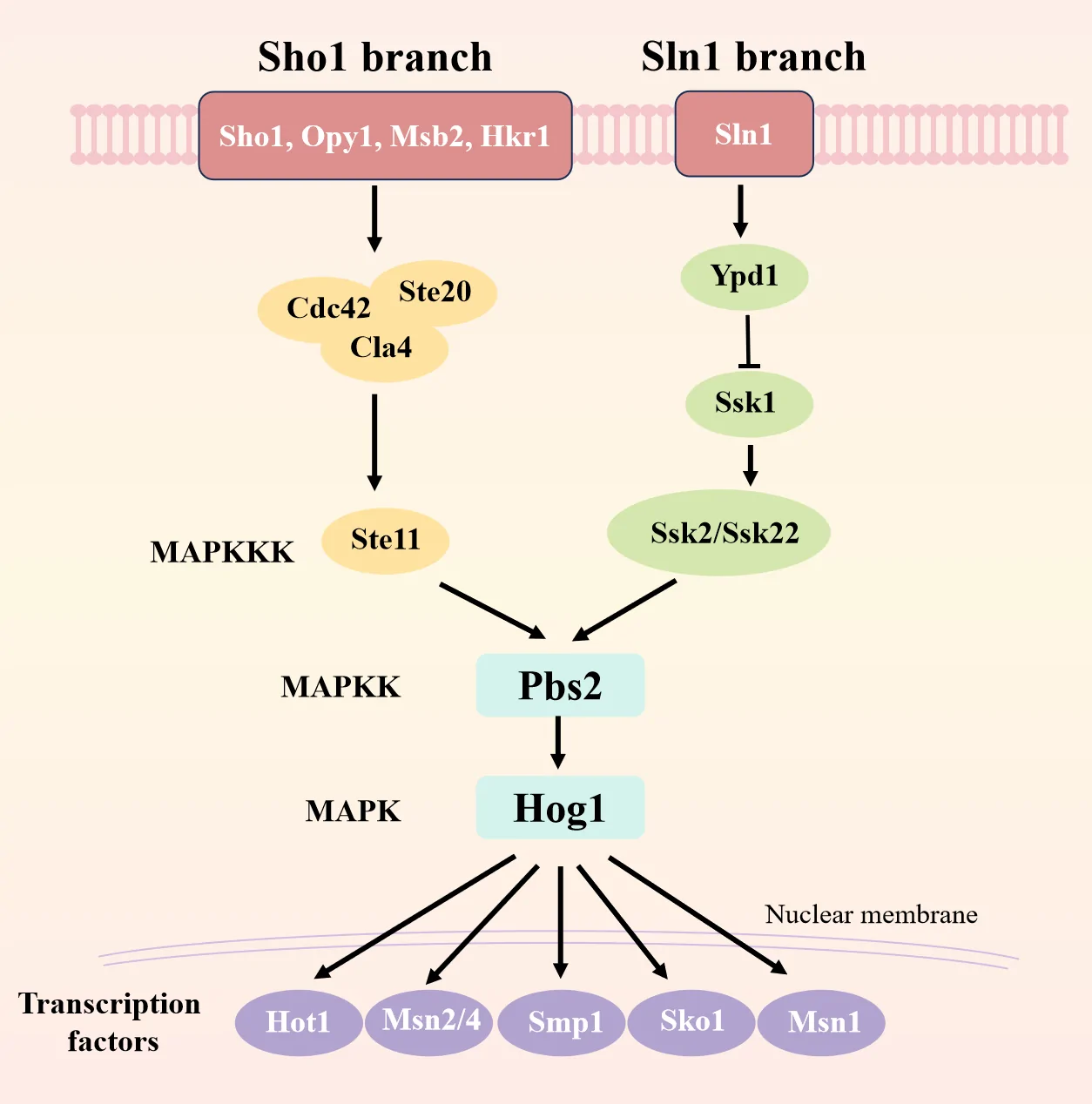
Schematic overview of the HOG MAPK signaling pathway in *S. cerevisiae*. Environmental stress signals are sensed by two independent plasma membrane branches: the Sho1 branch and the Sln1 phosphorelay branch. Signals from both branches converge on the shared MAPKK Pbs2, which activates the terminal MAPK Hog1. Upon nuclear translocation, Hog1 regulates multiple transcription factors to mediate stress-adaptive transcriptional responses. Arrows indicate activation, whereas the T-shaped bars represent inhibition. MAPKKK, mitogen-activated protein kinase kinase kinase; MAPKK, mitogen-activated protein kinase kinase; MAPK, mitogen-activated protein kinase.

The upstream of the HOG signaling pathway consists of two independent signaling branches, the Sln1 branch and the Sho1 branch, whose signals converge on the shared MAPKK Pbs2. The overall architecture of the HOG pathway, including its upstream sensing branches, core MAPK module, and downstream transcription factors, is depicted in Figure 1.

#### 2.2.1. The Sln1 Branch

The Sln1 branch employs the histidine kinase Sln1 as its signal sensor. Under normal growth conditions, Sln1 remains active and autophosphorylates on its histidine residue. These phosphate groups are sequentially transferred to Ypd1 and ultimately to the receiver domain of the response regulator Ssk1 [9,10]. In this phosphorylated state, Ssk1 adopts an inactive conformation and cannot initiate the downstream MAPK cascade reaction. Upon hyperosmotic stress, Sln1 kinase activity is inhibited, blocking its autophosphorylation and preventing the subsequent phosphorylation of Ypd1 and Ssk1. Dephosphorylated Ssk1 then binds to the N-terminal regulatory domains of the MAPKKKs Ssk2 and Ssk22, inducing their autophosphorylation and activation [11]. Activated Ssk2 and Ssk22 subsequently phosphorylate and activate the MAPKK Pbs2, which in turn activates the downstream MAPK Hog1 [10].

#### 2.2.2. The Sho1 Branch

The Sho1 branch employs two mucin-like transmembrane glycoproteins, Msb2 and Hkr1, which serve as osmotic stress sensors. These highly glycosylated single-pass transmembrane proteins perceive external stress signals and initiate downstream signaling [12,13]. The Sho1 branch can be further divided into Hkr1- and Msb2-dependent sub-branches, which activate the HOG pathway through distinct regulatory mechanisms. These sensors interact with the transmembrane protein Sho1 and bind to the small GTPase Cdc42, leading to the activation of Ste20 and Cla4. Activated Ste20/Cla4 subsequently phosphorylate and activate MAPKKK Ste11 [12]. Ste11 localizes to the cell membrane via the scaffold protein Ste50, with which it forms a stable complex. In this complex, Ste50 primarily binds the membrane-anchored protein Opy2, and secondarily interacts with Cdc42 and Sho1 to facilitate membrane localization [14]. Activated Ste11 subsequently phosphorylates and activates MAPKK Pbs2, which in turn phosphorylates and activates MAPK Hog1. Recent studies have defined the multi-layered regulatory logic of Sho1 branch signaling under hypertonic conditions. Tatebayashi and Saito demonstrated that stress regulates three distinct steps: it promotes Ste11-mediated phosphorylation of Pbs2 through Sho1-dependent interactions requiring Hkr1, and it also controls Ste11 activation that depends on Hkr1 and Opy2 [15]. Together with the Pbs2-mediated phosphorylation of Hog1, this stepwise multi-gate control ensures that Hog1 activation occurs specifically under hyperosmotic stress.

### 2.3. Branch-Specific Activation by Diverse Stressors

**Figure 2:**
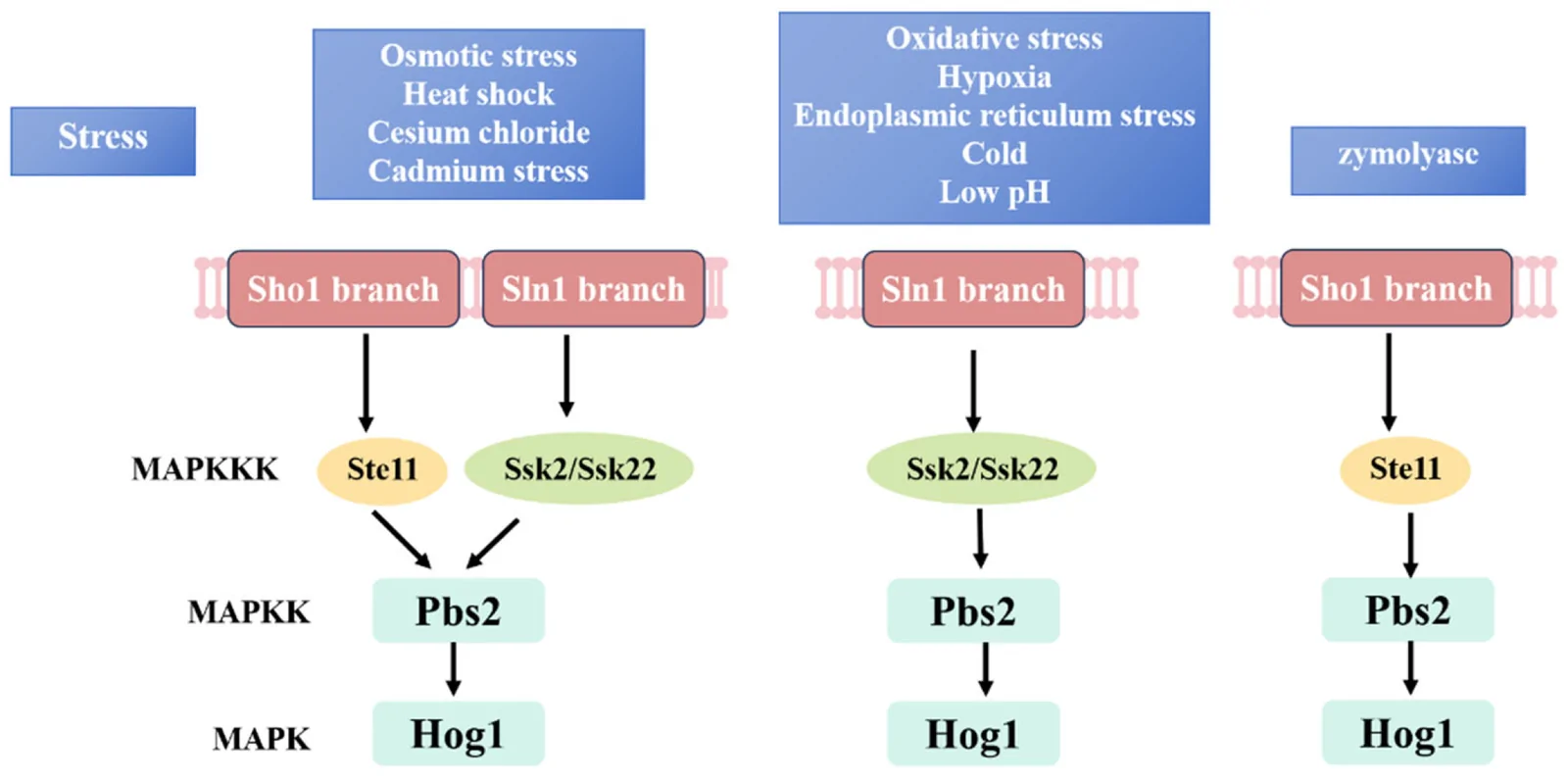
Stress-specific engagement of the Sho1 and Sln1 branches in HOG pathway activation. The Sln1 branch is engaged by oxidative stress, hypoxia, ER stress, cold, and low pH. The Sho1 branch is engaged by cell wall stress (zymolyase). Osmotic stress, heat shock, cadmium and cesium chloride stresses engage both branches to varying extents. Abbreviations: ER, endoplasmic reticulum; HOG, high-osmolarity glycerol. Arrows indicate activation. All schematics in this figure are original and were created by the authors.

Different types of stress engage distinct upstream signaling branches to activate the HOG pathway. This stress-specific branch selection is governed by the nature of the stress signal and the sensing capabilities of each branch, as summarized in Figure 2. The molecular mechanism by which osmotic stress activates the HOG pathway has been elucidated: the stress signal is transmitted through both the Sho1 and Sln1 branches, which exhibit functional compensation—when one branch is impaired, the other can compensate [16]. The Sho1 and Sln1 branches exhibit distinct activation thresholds: the Sln1 branch is activated even at low osmolarity, whereas the Sho1 branch is engaged mainly under severe stress conditions [16,17]. This threshold difference allows the HOG pathway to respond across a broad range of osmotic intensities, with the two branches providing complementary functions. Consistently, the two branches also differ in how they phosphorylate Pbs2: Ste11—the MAPKKK downstream of Sho1—phosphorylates only Thr518, whereas Ssk2/Ssk22—the MAPKKKs downstream of Sln1—phosphorylate both Ser514 and Thr518 [18]. Of note, the two phosphorylation sites (Ser514 and Thr518) of Pbs2 are differentially regulated by upstream kinases and are also differentially dephosphorylated by four PP2C phosphatases (Ptc1–Ptc4) [19]. Different phosphatases exhibit distinct dephosphorylation efficiencies for these two sites, thereby fine-tuning the activation intensity and duration of the HOG pathway under different stress conditions.

In addition to osmotic stress, several other environmental stresses—including cadmium stress, cesium chloride stress, and heat shock stress—also activate the HOG pathway. However, unlike osmotic stress, which engages both upstream branches, these non-osmotic stresses typically activate only one branch (Sln1 or Sho1), and the resulting Hog1 phosphorylation kinetics differ significantly from those observed under osmotic stress [16,17,20,21,22,23].

The Sln1 branch serves as the primary route for most non-osmotic signals, including oxidative stress, hypoxia, ER stress, cold, and low pH [24,25,26,27]. ER stress induced by tunicamycin requires Sln1 and Ssk1 (but not Ssk22) for HOG activation, and Hog1 phosphorylation is sustained even when the kinase is retained in the cytosol [26,28]. Cold stress activates Hog1 specifically through the Sln1 branch in response to decreased membrane fluidity. This activation drives glycerol accumulation and enhances freeze tolerance in a *HOG1*- and *GPD1*-dependent manner [29,30]. Low pH stress also activates HOG via Sln1, inducing Hog1-dependent cell wall reorganization, including enhanced resistance to β1,3-glucanase and altered anchoring of cell wall proteins, with transcriptional induction of Hog1-dependent genes such as *CWP1*, *HOR7*, *SPI1*, and *YGP1* [31,32].

In contrast, cell wall damage elicited by lytic enzymes (e.g., Zymolyase) activates the Sho1 branch without detectable input from the Sln1 phosphorelay [33]. Sho1 assembles a multi-kinase scaffold complex containing Ste11, Ste20 and Pbs2, leading to Hog1 phosphorylation that primarily coordinates cell wall remodeling through crosstalk with the CWI pathway.

A small subset of stressors, including cadmium exposure and cesium chloride, have been reported to engage both sensing branches to varying extents, whereas heat shock has been reported to activate the HOG pathway primarily through Sho1, although contradictory results exist regarding Sln1 involvement [22,23,34]. Unlike hyperosmotic treatment, this dual activation pattern does not trigger massive glycerol overproduction; instead, Hog1 primarily mediates metal detoxification and heat-induced chaperone expression under these conditions [35].

Notably, acetic acid activates Hog1 via an intracellular pathway independent of the Sln1 and Sho1 branches. Acetic acid induces cytoplasmic stress granules (SGs) that act as scaffolds to concentrate basal Pbs2 and Hog1, thereby driving Hog1 phosphorylation without upstream MAP3K stimulation [36]. This process relies on Sln1-sustained basal Pbs2 activity, whereas Sho1 plays no role here. Activated Hog1 is retained in SGs and cannot translocate to the nucleus, so glycerol biosynthesis genes are not induced. Instead, SG-localized phosphorylated Hog1 targets Fps1, dissociating its regulators Rgc1/Rgc2 to trigger channel endocytosis and reduce acetic acid uptake. This unique signaling mode distinguishes acetic acid from most other activators of the HOG pathway.

Collectively, the HOG pathway employs several modes of stress activation: dual-branch engagement, single-branch engagement through Sln1 or Sho1, and branch-independent activation via stress granules (acetic acid). These distinct modes correlate with specific Hog1 phosphorylation kinetics and downstream outputs, suggesting that branch selection influences response specificity by modulating Hog1 phosphorylation kinetics and transcriptional outputs.

## 3. Multifaceted Roles of the HOG Pathway in Stress Adaptation

Living in a dynamic environment, yeast undergoes various stresses such as hyperosmotic stress, oxidative stress, acid stress, ethanol stress, and heat shock. To cope with these stresses, cells have evolved multiple adaptive stress responses that enable survival and maintain homeostasis. In eukaryotic cells, activation of the HOG pathway initiates a set of adaptive responses, including regulation of gene expression, cell cycle arrest, modulation of intracellular osmolyte synthesis, and other specialized reactions.

### 3.1. Transcriptional Reprogramming: Global Control of Gene Expression

In response to sudden changes in environmental factors, cells must rapidly adjust their gene expression programs to adapt. Upon activation, Hog1 rapidly translocates to the nucleus and induces the expression of hundreds of stress-responsive genes [37,38]. It is generally accepted that under osmotic stress, these transcriptional changes fall into two categories: genes specifically induced by osmotic stress (e.g., *GPD1*), and genes that respond to diverse stress, collectively termed the Environmental Stress Response (ESR) [39]. The ESR comprises 300–600 genes that undergo transcriptional reprogramming (upregulation or downregulation) in response to stresses such as DNA damage, heat shock, osmotic stress, and oxidative stress [40]. Upregulated ESR genes are enriched in carbohydrate metabolism, protein metabolism, intracellular signal transduction, reactive oxygen species resistance, and DNA damage repair, whereas downregulated genes are primarily associated with protein synthesis and growth-related processes [41,42].

Hog1 regulates gene expression through multiple mechanisms, including direct phosphorylation of transcription factors, modulation of chromatin structure, and control of transcriptional elongation and mRNA processing [4]. Major transcription factors regulated by Hog1 contain both positive regulators (Hot1, Smp1, Msn1, Msn2, Msn4) and the repressive factor Sko1 [43,44,45]. Activated transcription factors (TFs) bind to their target promoters in a coordinated manner to mediate dynamic responses. Direct phosphorylation of promoter-specific transcription factors is the best-characterized mechanism by which Hog1 MAPK regulates transcriptional initiation. In vivo co-immunoprecipitation and phosphorylation studies have demonstrated physical interactions between Hog1 and both Smp1 and Sko1, alongside direct phosphorylation of the two substrates by activated Hog1 [44,46].

In addition to its role in transcriptional initiation, Hog1 also contributes to multiple steps of mRNA biogenesis, including chromatin remodeling, recruitment of RNA polymerase II (RNA Pol II) to target promoters, transcriptional elongation, and mRNA nuclear export [4,47]. For instance, the presence of Hog1 in coding regions is critical for increasing the association of RNA Pol II with these regions. Under osmotic stress, Hog1 interacts with RNA Pol II and components of the transcription elongation complex, indicating that Hog1 directly influences elongation [48].

Chromatin remodeling complexes and chromatin-modifying enzymes are central factors in stress-mediated gene expression, and a dynamic balance among different chromatin remodeling complexes appears to be essential for regulating stress-responsive genes. Additional research also shows that Hog1 affects mRNA stability, particularly that of genes upregulated under osmotic stress [4].

Collectively, Hog1 coordinates a multi-layered transcriptional program that extends beyond simple activation of stress-responsive genes. By integrating direct phosphorylation of transcription factors with co-transcriptional regulation of chromatin remodeling, RNA polymerase II recruitment, elongation, and mRNA processing, Hog1 ensures both the intensity and timing of the stress-induced transcriptome are precisely controlled.

### 3.2. Cell Cycle Arrest: Buying Time for Repair and Adaptation

**Figure 3:**
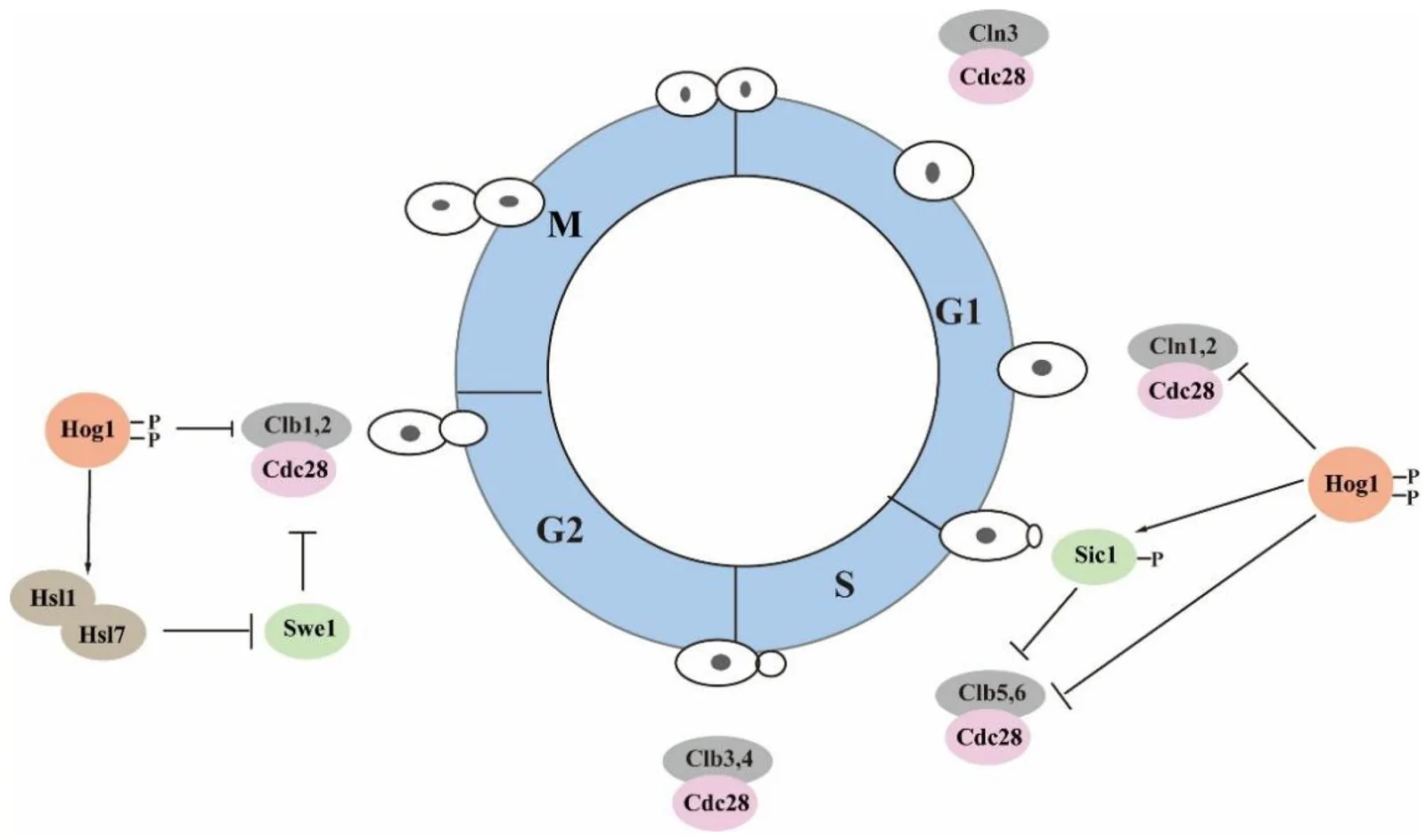
Hog1-mediated regulation of cell cycle checkpoints in *S. cerevisiae*. Activated Hog1 induces cell-cycle arrest at distinct phases by regulating cyclin-Cdc28 complexes and the inhibitory factors Sic1 and Swe1, coordinating cell proliferation with environmental stress responses. Arrows indicate activation; blunt-ended lines indicate inhibition.

Different stress responses exhibit significant differences in sensing and regulation mechanisms. However, an almost universal feature of cellular adaptation to stress in yeast is the slowing or arrest of the cell cycle, which reduces growth rates. This conserved response prevents genetic instability arising from erroneous DNA replication or chromosome segregation during stress, thereby safeguarding genomic integrity. Furthermore, arresting division diverts more intracellular energy toward stress responses, facilitating a more effective response [49,50]. Cell cycle control constitutes a well-characterized core canonical function of HOG signaling. In budding yeast, activated Hog1 enforces transient, reversible arrest at G1, S and G2/M checkpoints to create a time window for damage repair and stress reprogramming, with the full regulatory network illustrated in Figure 3 [50,51,52].

The G1 phase determines the initiation of a new cell division cycle. Under osmotic stress, Hog1 mediates a transient G1-to-S transition delay primarily through two mechanistically distinct but functionally coordinated pathways. The first and best-characterized mechanism involves the cyclin-dependent kinase (CDK) inhibitor Sic1. Upon activation, Hog1 directly phosphorylates Sic1 at Thr17, preventing its recognition by the E3 ligase Cdc4 and thereby blocking its ubiquitination and degradation [53]. Stabilized Sic1 then inhibits the Cdc28-Clb5,6 complex, which is required for S-phase entry, resulting in G1 arrest. This pathway is supported by direct biochemical evidence of Hog1–Sic1 interaction and site-specific phosphorylation [53,54]. The second mechanism operates at the transcriptional level: Hog1 downregulates the expression of G1 cyclins Cln1 and Cln2 by phosphorylating the transcriptional regulators Whi5 and Msa1, and also represses the S-phase cyclin *CLB5* [55,56]. Notably, these two pathways are not functionally redundant but temporally coordinated: in early G1, Hog1 primarily suppresses cyclin expression, whereas in late G1, as cyclin levels rise, Sic1 stabilization becomes the dominant barrier to S-phase entry [57]. The G1/S regulatory framework described above is not unique to osmotic stress. Beyond hyperosmotic stimuli, copper stress is also capable of triggering this Hog1-dependent G1 arrest. As reported by Ren et al., copper-derived oxidative stress activates Hog1 signaling and drives cell cycle arrest at the G1 phase [58].

In response to osmotic stress, Hog1 can impose S-phase arrest, but the underlying mechanism differs depending on when the stress is encountered during DNA replication. When cells are exposed to stress in early S phase, Hog1 delays the expression of the S-phase cyclins Clb5 and Clb6; in the late S phase, it instead interacts with replication complex components and delays the phosphorylation of the DNA polymerase subunit Dpb2 [51]. This arrest may serve to prevent replication from interfering with the transcription of stress-responsive genes and maintain genomic stability [4].

The G2/M transition requires activation of the Cdc28-Clb1,2 complex, whose activity is negatively regulated by the Swe1 kinase and positively restored by the Mih1 phosphatase [59]. Activated Hog1 induces G2 cell cycle delay through two mechanisms: it downregulates the mitotic cyclin Clb2 (though the underlying mechanism remains unidentified), and it reduces Clb2/Cdc28 activity via the Hsl1–Swe1 axis—Hog1 phosphorylates Hsl1, promoting Hsl7 dissociation from the bud neck, which leads to Swe1 accumulation and Clb2/Cdc28 inhibition [60,61].

Thus, Hog1 coordinates cell cycle progression with stress adaptation by targeting distinct regulators at each phase, ensuring genomic stability while allowing time for adaptive responses.

### 3.3. Metabolic Reprogramming: Glycerol as a Key Effector Molecule

Glycerol biosynthesis and homeostasis constitute one of the best-characterized core canonical functions of the HOG cascade, representing the primary adaptive output upon hyperosmotic stress. Rapid glycerol accumulation constitutes the defining cellular response to high osmolarity, as this compatible osmolyte protects intracellular macromolecules and maintains cellular osmotic homeostasis. The glycerol biosynthetic program is activated within one minute of stress exposure, with marked elevation of intracellular glycerol levels detected 30 min post hyperosmotic shock [62].

**Figure 4:**
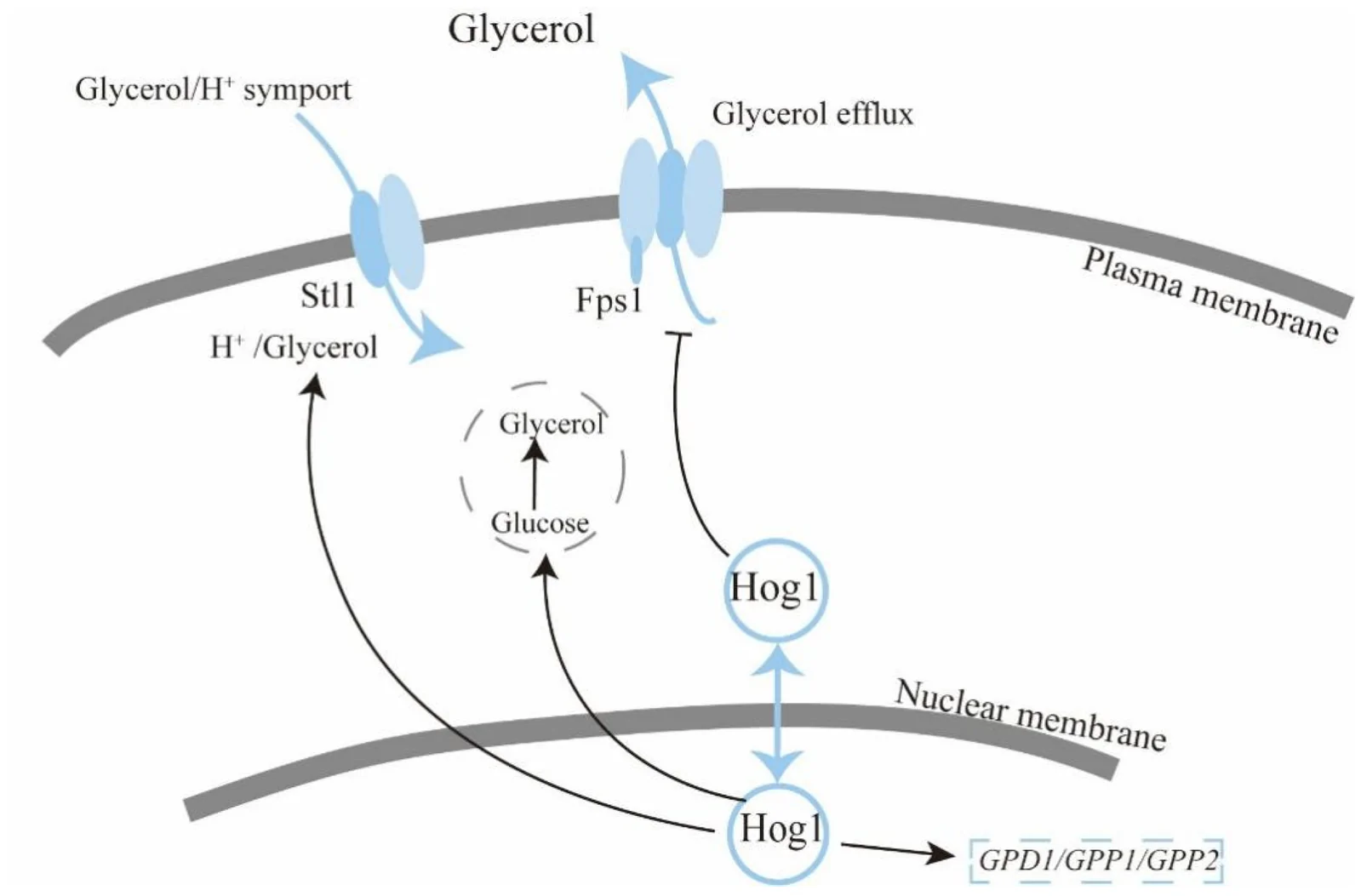
Hog1-governed glycerol homeostasis in *S. cerevisiae*. Activated Hog1 coordinates multiple processes to maintain intracellular glycerol pools: transcriptional induction of glycerol biosynthetic genes (*GPD1*, *GPP1*, *GPP2*), promotion of glycerol uptake via the Stl1 symporter, enhancement of glycolysis to supply glycerol precursors, and inhibition of the Fps1 channel to block glycerol efflux. Arrows indicate activation, whereas the T-shaped bars represent inhibition.

Extensive genetic, biochemical and systems biology studies have demonstrated that the HOG pathway acts as the master regulator of glycerol homeostasis, governing the entire metabolic chain including glycerol synthesis, extracellular uptake and intracellular efflux via four distinct but coordinated mechanisms [7]. First, Hog1 mediates transcriptional activation of glycerol biosynthetic genes. Upon stress-induced nuclear translocation, phosphorylated Hog1 activates the expression of *GPD1*, *GPP1* and *GPP2*. Second, Hog1 upregulates the expression of the H^+^-coupled active transporter Stl1, which mediates efficient glycerol uptake from the surrounding medium, serving as a supplementary pathway for intracellular glycerol accumulation. Third, Hog1 modulates glycolytic dynamics by activating 6-phosphofructo-2-kinase (Pfk26/27). The product fructose-2,6-bisphosphate is a potent allosteric activator of glycolysis, which accelerates the catabolism of glucose and generates abundant dihydroxyacetone phosphate (DHAP), the direct precursor for glycerol synthesis. Fourth, the aquaglyceroporin Fps1 is a major glycerol efflux channel. Under hyperosmotic stress, activated Hog1 phosphorylates the Fps1 regulators Rgc1 and Rgc2, causing them to detach from the channel, thereby reducing Fps1-mediated glycerol efflux and retaining intracellular osmolytes. These mechanisms are summarized in Figure 4.

Beyond its direct role in synthesis and accumulation, the HOG pathway also governs glycerol assimilation, exerting dual regulation of glycerol metabolism. Sone et al. demonstrated that loss of Hog1 activity enhances glycerol assimilation in *S. cerevisiae*, suggesting that Hog1 acts as a negative regulator of glycerol uptake or catabolism under physiological conditions [63]. This multi-layered regulator mechanism enables rapid glycerol accumulation upon stress exposure. Furthermore, Hog1-induced glycerol accumulation inhibits excessive accumulation of the small ubiquitin-like modifier (SUMO) under stress [6]. Multiple stress stimuli cause significantly increased SUMO accumulation, and excessive SUMO impairs stress adaptation [64,65]. Hog1 alleviates this toxic effect through transcriptional activation of glycerol biosynthesis genes, thereby reducing excessive SUMO conjugation [66,67].

The HOG pathway-mediated regulation of glycerol is not limited to hyperosmotic stress; diverse stress can also activate this pathway to promote glycerol synthesis, helping cells resist adverse environments [68,69]. Multiple studies confirm that HOG-mediated glycerol production protects yeast against heat and oxidative stress [68,69]. A heat stress study by Siderius et al. further verified that at 37 °C, reduced osmotic sensitivity of Hog1 mutants correlates with increased glycerol accumulation, highlighting the role of glycerol regulation in osmotic signaling under heat stress [69]. Additionally, Hog1 can enhance cellular tolerance to arsenite, acetic acid, and heat shock by regulating Fps1 [23,70,71,72]. In ER stress triggered by tunicamycin, Hog1 regulates *GPD1* transcription and promotes glycerol accumulation; knockout of glycerol synthesis genes drastically sensitizes yeast to tunicamycin toxicity [26]. Apart from glycerol, the synthesis of other osmolytes such as trehalose also helps cells restore osmotic balance under adverse conditions, and plays roles in antioxidant defense, cellular detoxification, and maintenance of protein conformation [73].

Collectively, the HOG pathway governs glycerol homeostasis by coordinating synthesis, uptake, efflux control, and assimilation. This regulatory framework not only ensures rapid osmolyte accumulation upon osmotic stress but also contributes to protection against diverse stresses such as heat shock, oxidative stress, and ER stress.

### 3.4. Coordination of Other Adaptive Responses

In addition to the well-characterized adaptive responses described above, the HOG pathway governs several other stress-related processes that extend its regulatory scope. These include cell wall remodeling, flocculation, mitophagy, and cross-talk with other MAPK pathways, collectively pointing to a broader role for Hog1 in coordinating diverse aspects of cellular stress adaptation [67].

#### 3.4.1. Coordination of Cell Wall Remodeling

Under environmental stress, the HOG pathway regulates cell surface composition by directly modulating cell wall components or through crosstalk with other signaling pathways, thereby enhancing stress resistance [22,74]. Hernández-Elvira et al. demonstrated that under tunicamycin-induced endoplasmic reticulum stress, Hog1 regulates multiple processes, including lipid and carbohydrate biosynthesis, protein glycosylation, vesicle-mediated transport, cell wall organization and biosynthesis, as well as cellular detoxification [75]. Two Hog1-regulated genes, *NAB6* and *ECM13*, were identified as key players in cell wall organization under this condition. The cell wall integrity (CWI) pathway is the primary signaling pathway responsible for mediating cell wall remodeling in yeast, and it typically cooperates with the HOG pathway to regulate cell wall synthesis [76]. Similarly, Vescovo et al. analyzed the overall transcriptional response of *S. cerevisiae* to different concentrations of cesium chloride. They found that early-response genes were primarily involved in cell wall structure and biosynthesis, and about half of these genes had been previously shown to respond to other alkali metal cations in a Hog1-dependent manner [35]. Furthermore, the cellular response to cesium was also found to involve the CWI pathway [22]. In addition, the cooperation between HOG and CWI pathways in regulating cell wall synthesis extends to various other stresses, including ethanol stress and low pH [31,74].

#### 3.4.2. Regulation of Flocculation

**Figure 5:**
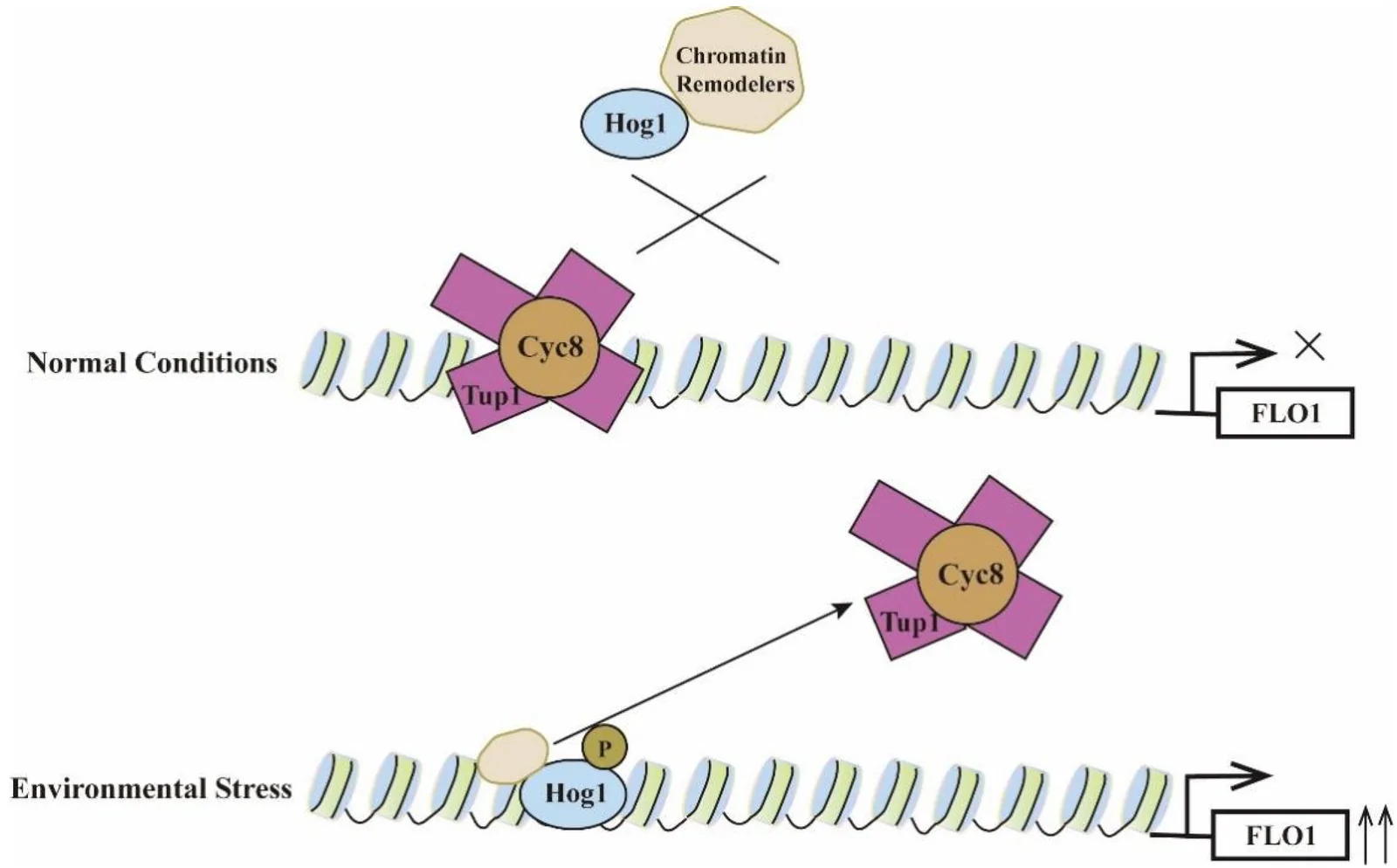
Diagram depicting the role of Hog1 MAPK in the de-repression of *FLO* genes expression. Under normal conditions, the *FLO* gene repressor complex Tup1-Cyc8 binds to *FLO* promoters, repressing *FLO* gene expression and consequently suppressing flocculation. Upon environmental stress, activated Hog1 translocates to the nucleus and binds to *FLO* promoters, reducing the affinity of the Tup1-Cyc8 complex and facilitating the formation of an active chromatin structure that promotes *FLO* gene expression and flocculation. Arrows indicate activation; the X marks (or crossed lines) indicate blockage, dissociation, or repressive effects; distinct colors are used to distinguish different proteins.

In addition to cell wall remodeling, the HOG pathway also regulates flocculation, a stress-induced survival mechanism in which yeast cells aggregate to withstand unfavorable conditions. Kumawat and Tomar demonstrated that under environmental stress, activation of the HOG pathway leads to Hog1 phosphorylation and nuclear translocation, where it binds to the promoters of *FLO* genes [77]. This binding reduces the affinity of the Tup1-Cyc8 repressor complex for the *FLO* promoter, likely facilitating the formation of an active chromatin structure that promotes *FLO* gene upregulation. Consistently, deletion of *HOG1* or pharmacological inhibition of Hog1 phosphorylation by cantharidin treatment results in the downregulation of *FLO* genes and impaired flocculation (Figure 5).

Further studies revealed that flocculation also enhances tolerance to specific stresses, such as acetic acid, in a Hog1-dependent manner. Ye et al. showed that in flocculating cells, Hog1 overexpression reduces reactive oxygen species (ROS) accumulation and elevates glutathione peroxidase (Gpx1) activity, thereby improving ethanol production under acetic acid stress [78]. In addition, Hog1 functionally associates with other kinases, including Akl1 and Rim15, and these interacting partners work synergistically to form a signaling network that links flocculation to broader stress responses. Collectively, these findings further extend the role of the HOG pathway in coordinating cell surface properties and stress adaptation.

#### 3.4.3. Signal Cross-Talk with Other MAPK Pathways

Beyond its direct functions, the HOG pathway contributes to cellular adaptation through cross-talk with other MAPK pathways [79]. Multiple MAPK pathways in yeast share common components, enabling coordinated regulation of distinct stress responses and integration of diverse environmental cues. Notably, the HOG and CWI pathways utilize overlapping scaffold proteins and regulatory nodes to fine-tune their outputs under combined stress conditions, ensuring mutual modulation of pathway activities and enhancing the robustness of cellular stress responses.

The HOG pathway also engages bidirectional cross-talk with the mating pathway via the shared MAPK Kss1. Intriguingly, the two upstream branches of HOG exert opposing effects on Kss1: Sho1 activates Kss1, while Sln1 limits its activation [80]. When signaling proceeds exclusively through the Sho1 branch, the HOG pathway induces mating-related genes in a Kss1-dependent manner; conversely, pheromone stimulation dampens HOG-mediated transcription. Thus, by differentially engaging its Sho1 and Sln1 branches, the HOG pathway not only controls its own osmotic outputs but also actively modulates mating pathway activity through shared components like Kss1, thereby enabling integrated responses to combined stresses.

#### 3.4.4. Regulation of Mitophagy

The HOG pathway enhances cellular stress resistance by maintaining organelle homeostasis through the regulation of mitophagy. Rather than directly phosphorylating Atg32, activated Hog1 and its upstream MAPKK Pbs2 act upstream of casein kinase 2 (CK2) to enhance its catalytic activity. CK2 subsequently phosphorylates the mitophagy receptor Atg32 at Ser114 and Ser119, triggering the assembly of the Atg32-Atg11 complex and initiating mitophagy [81,82,83]. Mitochondria are particularly susceptible to oxidative damage under adverse conditions, and impaired organelles produce excessive reactive oxygen species (ROS), which further aggravates oxidative damage [84,85]. By promoting Atg32-dependent mitophagy, the HOG pathway drives the selective clearance of dysfunctional mitochondria, thereby limiting ROS accumulation and preserving cellular integrity [85,86,87]. Notably, distinct from the CWI MAPK effector Slt2 that controls both mitophagy and pexophagy, Hog1 specifically governs mitochondrial turnover, highlighting the specialized roles of distinct MAPK cascades in selective autophagy.

In summary, the HOG pathway extends its regulatory reach beyond canonical osmoregulation to encompass cell wall remodeling, flocculation, cross-talk with other MAPK pathways, and mitophagy. These functions collectively position Hog1 as a broad coordinator of cellular stress adaptation, acting at multiple levels from cell surface to mitochondria.

## 4. Conclusions

Collectively, the HOG MAPK pathway in *S. cerevisiae* has transcended its initial role as a dedicated hyperosmotic stress response module to become a paradigm for understanding how eukaryotic cells integrate and respond to diverse environmental signals. Its sophisticated upstream sensing system, consisting of the Sln1 and Sho1 branches, enables cells to distinguish different stress signals and trigger branch-specific activation. Its biological functions can be clearly divided into two categories: well-characterized core activities and less-explored emergent regulatory roles. The core, firmly established functions of HOG signaling rest on decades of biochemical and genetic evidence, including: (1) genome-wide transcriptional reprogramming driven by nuclear Hog1 via multiple transcription factors and chromatin remodeling; (2) transient cell cycle arrest at G1/S and G2/M checkpoints, which preserves genomic integrity during stress; and (3) multilayered glycerol metabolic homeostasis coordinating synthesis, uptake, and efflux to maintain osmotic balance. Beyond these canonical adaptive responses, recent accumulating evidence indicates emergent, incompletely characterized functions, including coordinated cell wall remodeling via crosstalk with the CWI pathway, stress-induced flocculation through *FLO* gene de-repression, selective mitophagy to eliminate damaged mitochondria, and cross-talk with other MAPK pathways. In contrast to the clearly defined signaling framework for core osmotic adaptation, the molecular mechanisms and physiological relevance of these emerging functions under complex stress conditions remain incompletely defined.

Despite the substantial progress achieved to date, several fundamental questions concerning the HOG pathway remain to be addressed. First, the molecular mechanisms underlying the precise interpretation of diverse stress signals and the selective engagement of the upstream Sln1 and Sho1 branches remain incompletely defined. Second, extensive crosstalk between the HOG pathway and other core signaling cascades, including CWI, PKA and TOR pathways, requires systematic investigation. Additionally, the spatiotemporal dynamics of Hog1 activity across subcellular compartments remain largely uncharacterized. With the rapid advancement of experimental technologies, system biology approaches—including quantitative proteomics and single-cell analysis—will facilitate comprehensive mapping of the HOG-centric regulatory network. A deeper understanding of the HOG pathway may provide reliable molecular targets for rational strain engineering of stress-tolerant industrial yeast, thereby enhancing fermentation performance under adverse conditions.

## Data Availability

No new data were created or analyzed in this study. Data sharing is not applicable to this article.
